# 137 The role of link workers in ‘bridging the gap’ to community-based physical activity

**DOI:** 10.1093/eurpub/ckae114.027

**Published:** 2024-09-26

**Authors:** Megan O’Grady, Deirdre Connolly, Emer Barrett

**Affiliations:** Discipline of Physiotherapy, Trinity College Dublin, Ireland; Discipline of Occupational Therapy, Trinity College Dublin, Ireland; Discipline of Physiotherapy, Trinity College Dublin, Ireland

## Abstract

**Purpose:**

Promoting local, community-based physical activity (local PA) can reduce barriers to participation in physical activity (PA). Link workers (LW) can improve health and wellbeing by facilitating connections to community and voluntary services which include local PA. The purpose of this study was to investigate the role of LW in connecting community-dwelling adults to local PA.

**Methods:**

This abstract presents results from two studies. In study 1, a scoping review was conducted. Full-text peer and non-peer reviewed studies reporting on the processes of intermediaries when connecting individuals to local PA were considered for inclusion. In study 2, semi-structured interviews were conducted with 27 Irish LW, including local sports partnership officers and social prescribing link workers, to describe their practices and processes when connecting individuals to local PA. Interviews were analysed using qualitative content analysis.

**Results:**

Study 1 found that LW received referrals, conducted assessments, and followed up with individuals over time using several strategies to facilitate uptake of local PA. In both studies, LW received few referrals specifically to increase PA. LW in study 2 instead reported receiving referrals for psychosocial needs or to address pre-existing health issues. Despite this, they often connected people to local PA, recognising its ability to improve physical, mental, and social health. LW in study 2 also reported heterogeneous processes when facilitating connections to local PA but reported that knowledge of local services was a key component of the process. Their strategies included person-led practical and instrumental support and selecting local PA tailored to the person’s interests, preferences, and abilities.

**Conclusions:**

LW in Irish and international settings connect a large proportion of individuals who are referred to their services to PA despite receiving few referrals for this purpose. Further studies are needed to (i) determine the reason for lack of specific PA referrals, (ii) identify optimum levels of support for people with different health needs, and (iii) investigate the effects of the LW intervention on physical activity outcomes.

**Support/Funding Source:**

Supported by the Glennon Bursary, awarded by the Irish Society of Chartered Physiotherapists: January 2023 – January 2024.

